# Comparing adverse maternal outcomes among adolescent and adult women in North Wollo Zone governmental hospitals, northern Ethiopia

**DOI:** 10.3389/fgwh.2025.1336661

**Published:** 2025-04-10

**Authors:** Tadele Emagneneh, Chalie Mulugeta, Belay Susu, Negesse Belayneh, Delelegn Tsegaye

**Affiliations:** ^1^ Department of Midwifery, College of Health Sciences, Woldia University, Woldia, Ethiopia; ^2^ Department of Midwifery, College of Medicine and Health Sciences, School of Nursing and Midwifery, Wollo University, Dessie, Ethiopia

**Keywords:** adolescent pregnancy, adult pregnancy, adverse maternal outcome, government hospitals, Ethiopia

## Abstract

**Background:**

Adolescents differ from adults in biological, social, and psychological characteristics, which can impact maternal health outcomes during pregnancy and childbirth. Research suggests that adolescents are at a higher risk of experiencing adverse maternal outcomes. However, the extent to which these differences are driven by various mediating factors—such as biological, lifestyle, or socioeconomic conditions—remains unclear. This study aimed to compare adverse maternal outcomes between adolescent and adult mothers in public hospitals in North Wollo Zone, northern Ethiopia, while adjusting for potential confounders such as healthcare access, antenatal care, and pregnancy intentions.

**Methods:**

A comparative cross-sectional study was conducted from November 2022 to February 2023 involving 488 mothers. Data were collected through interviews and clinical chart reviews and then entered into EpiData version 4.6.6.0 and analyzed using SPSS version 26. Descriptive statistics were used to summarize the data, and logistic regression was applied to identify significant variables (*p* < 0.05). To correct for multiple testing, the false discovery rate method, using the Benjamini–Hochberg procedure, was applied with a threshold of adjusted *p*-values <0.1.

**Results:**

Adolescent mothers exhibited significantly higher rates of adverse outcomes compared to adult mothers, including preterm labor (7.0% vs. 2.0%, *p* = 0.04), antepartum hemorrhage (11.9% vs. 4.9%, *p* = 0.014), anemia (19.3% vs. 10.2%, *p* = 0.006), pregnancy-induced hypertension (11.9% vs. 7.0%, *p* = 0.047), malpresentation (9.0% vs. 2.5%, *p* = 0.008), cephalopelvic disproportion (6.1% vs. 1.2%, *p* = 0.046), major perineal tears (8.6% vs. 3.3%, *p* = 0.016), and cesarean delivery (16.0% vs. 9.8%, *p* = 0.04).

**Conclusion:**

Adolescent pregnancy was strongly linked to a range of adverse maternal outcomes, including preterm labor, antepartum hemorrhage, malpresentation, oligohydramnios, anemia, major perineal tears, and an increased likelihood of cesarean delivery. To mitigate these risks, it is crucial to implement targeted community and health facility-based interventions that focus on preventing adolescent pregnancies and addressing contributing factors, ultimately improving maternal health outcomes among adolescents.

## Introduction

Adolescence, typically defined as the period between the ages of 10 and 19 years, involves significant physical, mental, and emotional changes that require additional attention to meet developmental needs (1). Despite the societal acceptance of childbearing for those aged 18–19 years in many parts of Ethiopia and sub-Saharan Africa, adolescent mothers in this age group are still often perceived as less mature, particularly in some cultural contexts (1, 2).

Globally, approximately 16 million births occur annually to mothers aged 15–19 years, accounting for 11% of all births, with nearly 95% of these in low- and middle-income countries (LMICs) (3–5). While pregnancies and births among adolescents aged 10–14 years are uncommon in most countries, they vary considerably in sub-Saharan Africa, where the proportion ranges from 0.3% to 12% (1, 6). Adolescent mothers are at a higher risk of maternal morbidity and mortality (7). Adolescent pregnancy is associated with poor child health outcomes, such as stillbirths and under-5 mortality (8). Sub-Saharan Africa, including Ethiopia, faces some of the highest mortality rates linked to adolescent pregnancies (2, 3). In addition, adolescent pregnancy exacerbates social and economic issues, including school dropout and the perpetuation of intergenerational poverty (1).

Maternal health risks differ for younger adolescents (under 18 years) compared to older adolescents (18–19 years old). Younger adolescents face higher risks of complications such as anemia, obstructed labor, and maternal mortality. At the same time, some studies suggest that maternal outcomes for older adolescents may be comparable to those of adult mothers, especially in terms of reduced maternal mortality, cesarean delivery rates, and hypertensive disorders (8). This highlights the need for context-specific research to understand the complex interactions of biological, social, and healthcare factors influencing maternal outcomes.

Improving maternal and child health is a critical focus of global health initiatives, including the Sustainable Development Goals (SDGs). However, adolescent pregnancies pose a significant barrier to achieving these objectives. According to the World Health Organization (WHO) data from 2014, complications during pregnancy and childbirth are the second leading cause of death among girls aged 15–19 years worldwide (9). In low- and middle-income countries, where 99% of maternal deaths occur, these complications are the leading cause of death in this age group and contribute substantially to maternal mortality among women aged 15–49 years (10). Adolescent mothers are more likely to experience complications such as obstructed labor, anemia, antepartum hemorrhage (APH), urinary tract infections, and preterm labor compared to adult mothers (2, 11–14). In Ethiopia, more than 75% of adolescent pregnancies are unintended, contributing to unsafe abortions and complications such as obstetric fistula (15, 16).

Efforts by governmental and non-governmental organizations to reduce adolescent mortality in Africa, particularly in sub-Saharan Africa, have achieved only modest progress (10, 17). Adolescent pregnancy remains a significant contributor to severe maternal outcomes, including mortality, and poses long-term health risks, such as impaired physical and mental development, that can impact future generations (2).

Adolescent girls in Ethiopia constitute 20%–22% of the female population and contribute to 12.5% of all births (18). Although numerous studies have explored the prevalence and determinants of adolescent pregnancy and related reproductive health issues, there is a lack of comprehensive understanding of how maternal outcomes differ between adolescent and adult pregnancies (19, 20). Furthermore, research directly addressing the adverse effects of adolescent pregnancy is limited, with most studies relying on secondary data, which constrains the ability to control for confounding factors effectively (13).

This study aims to contribute to the limited understanding of the adverse maternal outcomes associated with adolescent pregnancies in Ethiopia by comparing the outcomes of adolescent mothers with adult mothers in northern Ethiopia. The findings will provide insights into the unique challenges faced by adolescent mothers and inform targeted interventions to address these risks, offering support to adolescents, parents, and key stakeholders in improving maternal health outcomes.

## Methods

### Study area

This study was conducted in six public hospitals in the North Wollo Zone of Amhara National Regional State, Ethiopia. The zone's administrative center, Woldya, is located 521 km from Addis Ababa and 372 km from Bahir Dar. According to the United Nations’ 2023 projections and the World Population Prospects (2017), Ethiopia has approximately 23 million adolescents. The proportion of female adolescents is estimated to range between 49.5% and 54.9%, reflecting a more balanced distribution of male and female adolescents in the population. A 2023 report from the North Wollo Zone Health Office recorded 49 pregnant adolescents under age 15 and 5,788 aged 15–19 years at their first antenatal care (ANC) visit. The hospitals—Woldya Comprehensive Specialized Hospital, Kobo, Mersa, Kidus Lalibela, Meket Shedo, and Wadila Primary Hospitals—operate around the clock, offering maternal, child health, and emergency services.

### Study design and study period

An institution-based comparative cross-sectional study was conducted among adolescent and adult mothers who had given birth at public hospitals in the North Wollo Zone from 20 November 2022 to 20 February 2023. Women aged 35 and older were excluded from the study due to the increased risk of adverse maternal outcomes associated with advanced maternal age pregnancies (21).

### Population

This study focused on mothers 10–34 years of age who gave birth at an estimated gestational age (GA) of 28 weeks or more in one of the six selected government hospitals in the North Wollo Zone, Ethiopia. The study population was restricted to this subgroup, making it representative only of mothers who met these criteria and not of all mothers in the region. Inclusion criteria were limited to those who gave birth at or after a GA of 28 weeks, while mothers with multiple pregnancies, those referred from other health facilities, or those with incomplete records were excluded from the study to minimize potential biases.

### Sample size determination and sampling procedure

#### Sample size determination

The sample size was calculated using Epi Info with a 95% confidence level, 80% power, a 1:1 adolescent-to-adult pregnancy ratio, and a 10% non-response rate. Based on previous studies, pregnancy-induced hypertension (PIH) required the largest sample size. After adjusting for non-responses, the final sample size was 488 (Table 1).

**Table 1:** Sample size determination to compare adverse maternal outcomes among adolescent and adult women in North Wollo Zone hospitals, northern Ethiopia.

Variable	Considerations					Final sample size including 10% non-response rate	
	Percentage of exposed adolescents	Percentage of non-exposed adults	Power	95% CI	COR		
1. Low birth weight	21.1	9.3	80	95	2.6	322	(22)
2. Anemia	11.0	3.0	80	95	3.9	366	(12)
3. PIH	11.3	4.2	80	95	2.91	444 + 44 = 488	(13)
4. Preterm labor	20	6	80	95	3.9	208	(4)

COR, crude odd ratio.

#### Sampling techniques

**Figure 1:**
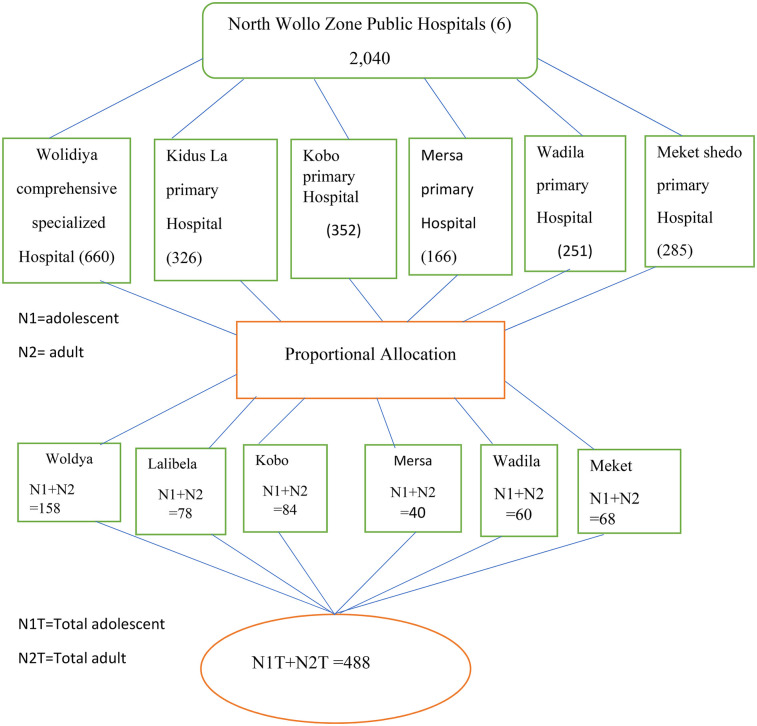
Show Schematic representation of sampling technique for adverse maternal outcomes between adolescent and adult women among mothers who came to labor and delivery in North Wollo Governmental Hospitals Northern Ethiopia 2022/2023.

A total of 488 participants were sampled and distributed proportionally across the six study hospitals: Woldya Comprehensive Specialized Hospital (158 participants), Kobo Primary Hospital (84), Mersa Primary Hospital (40), Kidus Lalibela Primary Hospital (78), Meket Shedo Primary Hospital (68), and Wadila Primary Hospital (60). The sampling process was based on a quota system, which was determined using the case reports from each hospital over the previous 3 months. Adolescent and adult mothers who met the inclusion criteria were consecutively interviewed from the start of data collection until the pre-determined sample quota was achieved. Given the higher number of adult births at these hospitals, a 1:1 ratio of adolescent to adult mothers was maintained through the downsampling of adult births. This downsampling was carried out randomly at each hospital to ensure that the adult birth sample remained representative of the total number of births at each site. This strategy helped balance the sample, conserve person-hours, and maintain the overall representativeness of the data (Figure 1).

### Variables

#### Variables of the study

The outcome variable was adverse maternal outcomes.

#### Independent variables

The independent variables included sociodemographic factors, such as maternal age, residence, religion, marital status, and the education levels of both the mother and husband and their occupations, and pregnancy-related characteristics including ANC, frequency of ANC, timing of initial ANC, and whether the pregnancy was planned or unplanned. In addition, variables related to medical conditions, self-medication practices, minor discomforts, and the onset of labor were also considered.

### Operational definitions of the study

#### Adolescent pregnancy

Adolescent pregnancy refers to those mothers whose age was 10–19 years at the time of admission to the obstetrics ward after 28 weeks of gestation for delivery services (1).

#### Adult age pregnancy

Adult age pregnancy refers those mothers whose age was in the range of 20–34 years at the time of admission to the obstetric ward after 28 weeks of gestation for delivery services (21, 22).

#### Adverse maternal outcomes

In this study, adverse maternal outcomes refers to at least one of the following: anemia, premature rupture of membranes (PROM), preterm labor, malpresentation, cephalopelvic disproportion (CPD), obstructed labor, cesarean section (C/s), PIH, APH, polyhydramnios, oligohydramnios, major perineal tears, and postpartum hemorrhage (PPH). These outcomes were identified after 28 completed weeks and were diagnosed by healthcare professionals and confirmed by reviewing the patients’ medical cards (23). These outcomes were selected due to their high prevalence, impact on maternal and neonatal health in low-resource settings, and feasibility for data collection. Other outcomes, such as congenital anomalies, were excluded due to data limitations.

#### Major perineal laceration/tears

A major perineal laceration/tear refers to a third- or fourth-degree perineal laceration that is diagnosed by a healthcare professional (24).

#### Self-medication practice

Self-medication practice refers to a woman taking at least one modern/traditional medicine for self-diagnosed ailments/diseases during her current pregnancy without a prescription from a physician (25).

#### The presence of minor discomforts during pregnancy

The presence of minor discomforts during pregnancy refers to a woman having at least one mild ailment, such as varicose veins, fatigue, ptyalism, nausea, vomiting, backache, and cramps (12).

### Data collection tool and procedure

Data were collected using structured paper-based questionnaires adapted from the literature (9, 24–26) and revised to better align with the local context and study objectives. We clarified the questions posed to participants during data collection, focusing on adverse maternal outcomes. Participants were asked whether they experienced specific adverse outcomes (“yes” or “no”), which were further specified through interviews and a review of patients’ medical records. These outcomes included events occurring during pregnancy, labor, and the postpartum period, such as PIH, anemia, PROM, preterm labor, malpresentation, CPD, obstructed labor, C/S, APH, polyhydramnios, oligohydramnios, major perineal tears, and PPH, among others. Response options were typically binary (“yes” or “no”) or categorical where applicable.

Six diploma-holding midwives were hired as data collectors, and six bachelor's-degree midwives served as supervisors at the study sites. All mothers aged 10–34 years giving birth at the hospitals were assessed for eligibility. Eligible participants were interviewed and their clinical records were reviewed until the required sample sizes for each group were met. Data from mothers with normal vaginal deliveries were collected 1–2 h postpartum, while those with cesarean sections or complicated deliveries were interviewed once fully awake.

### Data quality control

Data quality control involved 1 day of training for data collectors and supervisors on the study's objectives, methods, ethical considerations, and the questionnaire. A pretest with 26 (5%) mothers at Hayk Primary Hospital assessed consistency, interview duration, and question clarity. Based on the pretest, the final Amharic questionnaire was used. Participants were informed about the study's goals and procedures. Supervisors and the principal investigator reviewed data daily for completeness. A subset of records was double-entered, discrepancies were resolved, and EpiData's validation features ensured accuracy through regular supervision.

### Data processing and analysis

After coding, verifying, and cleaning the data, it was imported into EpiData version 4.6.0.6 and exported to SPSS version 26 for analysis. Categorical variables were recorded, and complications during pregnancy, childbirth, and the postpartum period were initially categorized as adverse maternal outcomes (“yes” or “no”) and later grouped into specific categories (e.g., pregnancy-induced hypertension, post-term pregnancy, preterm labor, APH, or PPH) for easier analysis. Descriptive statistics, including frequencies, means, standard deviations (SD), and percentages, were used to summarize the sociodemographic and other characteristics of the study population and presented in tables. Dichotomous outcomes (“yes” or “no”) were also recorded for logistic regression analysis. Variables were categorized and analyzed using logistic regression to assess associations between maternal age and specific adverse maternal outcomes, adjusting for sociodemographic, obstetric, and healthcare access factors. Due to small sample sizes, exact logistic regression (using Stata 17) was applied, which is appropriate for handling small sample sizes in multivariable models. Multicollinearity among the independent variables was assessed, and the Hosmer–Lemeshow goodness-of-fit test was used to validate the model. Variables with a *p*-value <0.25 in the bivariable analysis were included in multivariable logistic regression. Separate logistic regression models were applied to different maternal outcomes and the results are presented. Adjusted odds ratios (AORs) and 95% confidence intervals (CIs) were used to examine associations between adolescent pregnancy and other explanatory variables. Statistically significant variables were identified with a *p*-value <0.05 and a 95% CI. To correct for multiple testing, the false discovery rate (FDR) method using the Benjamini–Hochberg procedure was applied, with adjusted *p*-values set at a threshold of <0.1.

### Ethical considerations

The study adhered to the Declaration of Helsinki and was approved by the Ethical Committee of Wollo University College of Medicine and Health Science (Ref. No. CMHS1354/13/15). Eligible mothers in the study area were invited to participate through interviews and access to medical records, with written informed consent obtained from all participants. Adolescents aged 18 years or older provided their consent, while those under 18 required consent from their legal guardians. Individuals without guardians were classified as non-respondents. Data were handled with strict confidentiality throughout the study.

## Results

### Sociodemographic characteristics

Data were collected from a total of 488 women, with 244 in the adolescent group and 244 in the adult group, across six public hospitals, achieving a 100% response rate. The average age (±SD) of the respondents was 17.90 (±1.043) years for the adolescents and 26.45 (±3.61) years for the adults. Among the adolescents, the youngest was 15 years old, comprising 2% (5) of the adolescent group. Over half of the adolescents (58.6%) and adult women (51.6%) lived in rural areas, and a significant proportion of these mothers were followers of the Orthodox religion (62.7% of the adolescents and 77.5% of the adults) (Table 2). In both groups, more than three-fourths of the women attended school (97.5% of the adolescents and 95.4% of the adults). Among those who were educated, 5.3% of the adolescent mothers and 22.1% of the adult mothers had attended college or higher education. A higher proportion of the adolescent mothers were unmarried compared to the adult mothers (59.3% vs. 13.1%). In addition, a greater proportion of the adolescent mothers were students (45.9% vs. 5.7%) and labor workers compared to the adult mothers (8.2% vs. 0.8%) (Table 2).

**Table 2:** Sociodemographic characteristics of the adolescent and adult mothers who gave birth in public hospitals in the North Wollo Zone, northern Ethiopia, between 20 November 2022 and 20 February 2023.

Variable	Adolescents (244)	Adults (244)	Total (488)
Residence			
Rural	143 (58.6%)	126 (51.6%)	269 (55.1%)
Urban	101 (41.4%)	118 (48.4%)	219 (44.9%)
Religion			
Orthodox	153 (62.7%)	189 (77.5%)	342 (70.1)
Muslim	84 (34.4%)	49 (20.1%)	133 (27.3%)
Protestant	7 (2.9%)	6 (2.5%)	13 (2.6%)
Marital status			
Married	72 (29.5%)	208 (85.2%)	279 (57.2%)
Single	141 (57.8%)	10 (4.1%)	152 (31.1%)
Divorced	28 (11.5%)	17 (7.0%)	45 (9.2%)
Widowed	3 (1.2%)	9 (3.7%)	12 (2.5%)
Mothers’ education level			
No formal education	52 (%)	83 (4.5%)	135 (3.5%)
Primary education	95 (38.9%)	42 (17.2%)	137 (28.1%)
Secondary education	84 (34.4%)	65 (26.6%)	149 (30.5%)
Tertiary	13 (5.3%)	54 (22.1%)	67 (13.7%)
Occupation of the mother			
Student	118 (48.4%)	14 (5.7%)	132 (27.0%)
House wife	26 (10.7%)	72 (29.5%)	98 (20.1%)
Farmer	65 (26.6%)	92 (37.7%)	157 (32.2%)
Government employed	2 (0.8%)	26 (10.7%)	28 (5.7%)
Privately employed	12 (4.9%)	28 (11.5%)	40 (8.2%)
Merchant	0	10 (4.1%)	22 (4.5%)
Other (labor worker)	21 (8.6%)	2 (0.8%)	23 (4.7%)
Husband’s education			
No formal education	32 (44.5%)	64 (30.8%)	96 (34.3%)
Primary education	16 (22.2%)	36 (17.3%)	52 (18.6%)
Secondary education	9 (12.5%)	35 (16.8%)	44 (15.7%)
Tertiary education	15 (20.8%)	73 (35.1%)	88 (31.4%)
Husband’s occupation			
Farmer	38 (52.8%)	74 (35.6%)	112 (40%)
Government employed	5 (6.9%)	35 (16.8%)	40 (14.3%)
Privately employed	10 (13.9%)	60 (28.8%)	70 (25%)
Merchant	12 (16.7%)	36 (17.3%)	48 (17.1%)
Other (labor workers)	7 (9.7%)	3 (1.5%)	10 (3.6%)

Other occupations = labor workers.

### Obstetric profile of the study participants

The average (±SD) gestational age for the adolescent mothers was 38.74 (±2.17) weeks, while for the adult mothers, it was 39.27 (±1.77) weeks. The mean birth weight (±SD) for the adolescent and adult mothers was 2,568.48 (±370.30) g and 2,972.05 (±405.45) g, respectively. A larger proportion of the adolescent mothers (over 75%) were primigravida compared to the adult mothers (around 50%), with 224 (91.8%) adolescents and 136 (55.7%) adults being first-time mothers. More than 50% of the adolescent mothers did not receive iron folate supplementation (132, 54.1%) compared to less than 20% of the adult mothers (28, 11.5%). In addition, over 50% of the adolescent mothers had no ANC visits (127, 52.0%) compared to less than 25% of the adult mothers (32, 13.1%). In total, 72.5% of the adolescent mothers (177) had experienced an unplanned pregnancy compared to one-third of the adult mothers (75, 30.7%) (Table 3).

**Table 3:** Obstetric profile and pregnancy characteristics of adolescent and adult mothers who gave birth in public hospitals in the North Wollo Zone, northern Ethiopia, between 20 November 2022 and 20 February 2023.

Variable	Adolescents (15–19 years) (*n* = 244)	Adults (20–34 years) (*n* = 244)	Total (*n* = 488)
Gravida			
Primigravida	218 (89.3%)	136 (55.7%)	354 (72.5%)
Parity			
Primipara	232 (95.1%)	151 (61.9%)	383 (78.5%)
Past pregnancy complications	11 (4.5%)	32 (13.1%)	43 (8.8%)
Medical illnesses in this pregnancy	26 (10.7%)	30 (12.3%)	56 (11.5%)
Self-medication practice during pregnancy	51 (20.9%)	48 (19.7%)	99 (20.3%)
Antenatal care (ANC) visit			
At least one ANC visit	117 (48.0%)	212 (86.9%)	329 (67.4%)
Gestational age at first booking (weeks)			
≤16	63 (53.8%)	158 (74.5%)	221 (67.2%)
>16	54 (46.2%)	54 (25.5%)	108 (32.8%)
Number of ANC visits			
≥4 visits	67 (60%)	138 (65.1%)	205 (62.3%)
Iron folate supplementation			
Iron folate taken	112 (45.9%)	216 (88.5%)	328 (67.2%)
Duration ≥3 months	51 (45.5%)	141 (65.3%)	192 (58.5%)
Place of last delivery			
Health institution	12 (85.7%)	90 (95.7%)	102 (94.4%)
Mode of last delivery			
Spontaneous vaginal delivery	9 (75.0%)	69 (76.7%)	78 (76.5%)
With forceps/vacuum	2 (16.7%)	8 (8.9%)	10 (9.8%)
Cesarean section	1 (8.3%)	13 (14.4%)	14 (13.7%)

### Factors associated with adverse maternal outcomes

In addition to age, the primary independent variable in this study, several factors were significantly associated with adverse obstetrical outcomes among the adolescent mothers. These factors included living in a rural area, experiencing an unplanned pregnancy, having a medical disorder during pregnancy, and not receiving ANC. For adult mothers, the significant factors were being multigravida, not having ANC, medical illness, and unplanned pregnancy. Notably, residence was a significant factor exclusively among the adolescent mothers (Table 4).

**Table 4:** Bivariable and multivariable logistic regression of adverse maternal outcomes between adolescent and adult mothers who gave birth in public hospitals in the North Wollo Zone between 20 November 2022 and 20 February 2023.

Group	Mean ± SD	COR	AOR (95% CI), *p*-value				
Adolescents	17.4 ± 0.17	0.58	0.50 (0.35–0.70), <0.001**				
Adults	26.4 ± 3.7	0.99	0.97 (0.87–1.09), 0.63				
Other variables							
Variable	Category	Adolescents (yes/no)	COR	AOR (95% CI), *p*-value	Adults (yes/no)	COR	AOR (95% CI), *p*-value
Residence	Urban	30/71	1	1 (reference)	20/98	1	
	Rural	64/79	1.92	1.87 (1.01–3.46), 0.046*	31/95	1.59	1.5 (0.77–2.89), 0.23
Marital status	Married	24/48	1	1 (reference)	41/166	1	1
	Single	58/83	1.40	0.85 (0.41–1.78), 0.67	4/7	2.31	1.82 (0.46–7.17), 0.39
	Widowed	1/2	1.00	1.14 (0.09–15.11), 0.92	2/7	1.6	1.16 (0.21–6.48) 0.87
	Divorced	11/17	1.29	1.61 (0.57–4.51), 0.37	4/13	1.25	1.11 (0.30–4.01), 0.88
Medical illness	Yes	13/81	1.69	2.56 (1.02–6.38), 0.04*	11/19	2.52	3.0 (1.31–7.18), 0.01*
	No	13/137	1	1 (reference)	40/174	1	1
Self-medication	Yes	28/33	1.50	1.61 (0.82–3.17), 0.17	15/38	1.5	1.399 (0.65–2.98), 0.39
	No	66/117	1	1 (reference)	36/155	1	1
Gravida	Multigravida	13/81	1.69	0.43 (0.17–1.10), 0.08	28/80	1	1
	Primigravida	13/137	1	1 (reference)	23/113	1.72	2.05 (1.09–4.14), 0.03*
Parity	Multipara	7/87	2.33	0.88 (0.09–2.28), 0.35	22/71	1	0.43 (0.15–1.22),0.11
	Primipara	5/145	1	1 (reference)	29/122	1	1
Contraceptive	Yes	67/110	0.78	1.479 (0.74–2.89), 0.27	41/162	0.79	0.77 (0.32–1.86), 0.57
	No	30/40	1	1 (reference)	10/31	1	
Intended pregnancy	Yes	16/51	1	1 (reference)	37/165	1	1
	No	78/99	2.51	2.31 (1.15–4.63), 0.02*	14/28	2.23	2.2 (1.09–5.26), 0.03*
Have ANC	Yes	35/82	1	1 (reference)	38/170	1	1
	No	59/68	2.03	2.03 (1.17–3.51), 0.01*	13/23	2.53	2.73 (1.2–6.13), 0.015*
Taking Iron/Folate	Yes	34/78	1	1 (reference)	37/162	1	1
	No	60/72	1.91	0.89 (0.31–2.54), 0.83	14/31	1.98	0.92 (0.19–4.44), 0.92
Minor discomforts	Yes	56/72	1.60	1.66 (0.93–2.99), 0.09	27/90	0.78	1.001 (0.5–1.99), 0.99
	No	38/78	1	1 (reference)	24/103	1	1

*Significant variables at *p*-value of ≤0.05. **Significance at *p*-value of ≤0.001.

### Age effect

The Hosmer–Lemeshow goodness-of-fit test result was 0.81 and 0.82 for the adolescents and adults, respectively.

### Adverse maternal outcomes among the adolescent and adult mothers

The adolescent mothers had a higher overall rate of adverse maternal outcomes compared to the adult mothers (38.5% vs. 20.9%, *p* = 0.01, AOR = 1.944, 95% CI: 1.164–3.247). Logistic regression revealed significantly higher rates of complications among adolescent mothers, including mild anemia, preterm labor, oligohydramnios, APH, malpresentation, CPD, cesarean delivery, major perineal tear, and PIH. Uterine rupture and hysterectomy occurred in 0.8% of the adolescent mothers, but neither occurred in the adult mothers. Anemia (22.5%) was more common among the adolescents, while postpartum hemorrhage (11.5%) was the most frequent adverse outcome in the adults. No maternal deaths were reported in either group (Table 5).

**Table 5:** Bivariable and multivariable analyses of adverse maternal outcomes between adolescent and adult mothers who gave birth in public hospitals in the North Wollo Zone between 20 November 2022 and 20 February 2023.

Adverse maternal outcome	Adolescents (*n* = 244)	Adults (*n* = 244)	COR	AOR	95% CI	*p*-value	Adjusted *p*-value (FDR)
Total adverse maternal outcomes	94 (38.5%)	51 (20.9%)	2.37	1.94	1.16–3.25	0.011*	–
Preterm labor	17 (7.0%)	5 (2%)	3.58	3.45	1.04–8.94	0.04*	0.062***
Premature rupture of membranes	8 (3.3%)	18 (7.4%)	0.43	0.42	0.18–0.998	0.09	0.1145***
Polyhydramnios	7 (2.9%)	17 (7%)	0.39	0.38	0.134–0.85	0.02*	0.047***
Oligohydramnios	37 (15.2%)	17 (7.0%)	2.4	2.38	1.304–4.37	0.005*	0.042***
Antepartum hemorrhage	29 (11.9%)	12 (4.9%)	2.61	2.41	1.19–4.89	0.014*	0.045***
Malpresentation	22 (9.0)	6 (2.5%)	3.93	3.6	1.393–9.33	0.008*	0.042***
Anemia	55 (22.5%)	28 (11.5%)	2.37	1.832	1.165–2.88	0.009	0.042***
Post-term pregnancy	12 (4.9%)	10 (4.1%)	1.21	0.91	0.339–2.46	0.85	0.915***
Cesarean delivery	39 (16.0%)	24 (9.8%)	1.74	1.70	1.013–3.0	0.04*	0.062***
Pregnancy-induced hypertension	29 (11.9%)	17 (7.0%)	2.86	2.19	1.01–4.77	0.047*	0.066***
Cephalopelvic disproportion	15 (6.1%)	3 (1.2%)	5.20	3.8	1.03–14.39	0 0.04*	0.062***
Postpartum hemorrhage	21 (8.6%)	28 (11.5%)	0.726	0.594	0.30–1.177	1.135	1.000
Major perineal tear	21 (8.6%)	8 (3.3%)	2.91	2.68	1.206–6.40	0.016*	0.045***
Urinary tract infection	34 (13.9%)	11 (4.5%)	3.43	1.76	0.785–3.93	0.17	0.198

AOR, adjusted odds ratio; 95% CI, 95% confidence level.

Hosmer–Lemeshow goodness-of-fit test = 0.62–0.84.

* Significance at *p*-value of ≤0.05.

** Significance at *p*-value ≤0.001.

*** Significant at adjusted *p*-value <0.1 by FDR (false discovery rate; using Benjamini–Hochberg procedure).

The model was adjusted for different factors, including residence, school attendance, marital status, medical illness, presence of minor discomforts in pregnancy, self-medication practice during the current pregnancy, intended/unintended pregnancy, ANC, and iron folate supplementation during the current pregnancy.

## Discussion

Adolescent pregnancy presents significant risks to both maternal and child health. Children born to adolescent mothers face elevated risks of stillbirth, neonatal mortality, and under-5 mortality, with these risks escalating as maternal age decreases. Socioeconomic challenges, such as limited resources, disrupted education, and financial hardship, further exacerbate these outcomes. A comprehensive approach is needed to address adolescent pregnancy, focusing on maternal and child health, social support systems, and access to education and reproductive health services.

Our study found that adolescent mothers experience nearly twice the rate of adverse maternal outcomes (38.5%) compared to adult mothers (20.9%), consistent with findings from northeast Russia (11) but higher than global averages (9) and studies from Turkey (26) and Nepal (27). These discrepancies may be due to variations in socioeconomic, cultural, healthcare, and biological factors across settings (28).

Adverse maternal outcomes, including preterm labor, PROM, PIH, oligohydramnios, major perineal tears, APH, CPD, malpresentation, cesarean delivery, and anemia, were significantly more common among adolescent mothers. Preterm labor, in particular, occurred at a rate of 17.0% in the adolescents compared to 2.0% in the adults, indicating a threefold higher risk. This higher prevalence may be attributed to underdeveloped uterine and cervical blood supply in adolescents, leading to subclinical infections and increased prostaglandin synthesis, which heighten the risk of preterm birth (29, 30). In addition, socioeconomic stressors may contribute to the mental instability of adolescent mothers, further increasing the risk of preterm labor (29). Similar findings have been observed in studies from Asmara (31), India, and Japan (32, 33), which may be attributed to comparable sample sizes and shared study settings with similar and common biological risks associated with adolescent pregnancies. However, research from Russia (11) reported differing results, possibly due to better healthcare quality and accessibility in that context.

Polyhydramnios was less common in the adolescent mothers (2.9%) compared to the adult mothers (7.0%). The higher prevalence in the adult mothers may be linked to multiparity, a well-known risk factor due to uterine distension and conditions such as gestational diabetes or fetal anomalies. In contrast, adolescent mothers are more likely to be primiparous, which may explain the difference (34). However, a study in Turkey (35) found no significant difference between the two groups. Oligohydramnios, however, was 2.38 times more common in the adolescent mothers, likely due to their physiological immaturity, which impairs their ability to adapt to pregnancy changes. Factors such as underdeveloped uterine and placental structures, hormonal imbalances, and inadequate nutrition may contribute to this outcome (36). This finding contrasts with research from Turkey (35), where no significant difference was found.

The adolescent mothers experienced APH complications at twice the rate of adult mothers (11.9% vs. 4.9%). This can be attributed to their physiological immaturity, which increases the likelihood of underdeveloped uterine and placental structures and the risk of complications such as placental abruption. Nutritional deficiencies and increased risk of PIH further contribute to these outcomes (34). This finding aligns with studies from Saudi Arabia (37) and India (32). However, this result contrasts with research from Tigray, Ethiopia (38), which reported that adolescents were significantly less likely to experience APH compared to adults.

The adolescent pregnancies were also more likely to be complicated by PIH, with a prevalence of 11.9% compared to 4.5% in the adults. This increased risk may stem from social stigma, partner violence, and the extreme youth of mothers under 20, which are associated with the development of PIH (39). Our finding aligns with studies from India (32), Pakistan (40), Saudi Arabia (37), southwest Ethiopia, Kenya, and India (13, 27, 32). However, this result contradicts studies from Nepal (23) and Asmara (31), which found no significant difference between the two groups. Variations in healthcare access, socioeconomic conditions, and cultural contexts may explain these discrepancies.

The adolescent mothers were 3.6 times more likely to experience malpresentation during labor than the adult mothers (22.0% vs. 6.5%). This increased risk may be due to factors such as primigravity, physical immaturity, and anatomical constraints, including underdeveloped pelvic structures (41). Similar results were observed in Iran (39), but studies from Turkey and Eritrea (31, 35) did not find significant differences.

The adolescent mothers experience a significantly higher rate of CPD during labor compared to the adult mothers (6.1% vs. 1.2%), primarily due to their ongoing physical development, smaller pelvises, shorter stature, nutritional deficiencies, limited prenatal care, and higher rates of preterm births leading to abnormal fetal positioning (36). This finding, consistent with studies in regions’ global averages (7) and countries including India (33), Nepal (21), and Eretria (32), highlights CPD as a common concern among adolescent mothers globally, driven by shared underlying factors.

Our findings revealed a significantly higher prevalence of anemia among the adolescent mothers (22.5%) compared to the adult mothers (11.5%). This disparity may stem from increased nutritional demands, lower iron stores, limited access to iron-rich foods, and poor health behaviors, compounded by inadequate prenatal care (42). These results align with a study from Asmara (32) but differ from an Iraqi study (41), likely due to variations in dietary habits, healthcare access, and socioeconomic conditions or differences in study methodologies and anemia definitions.

Cesarean deliveries were more common in the adolescent mothers (16.0%) compared to the adult mothers (9.8%), likely due to the higher incidence of labor complications and underdeveloped pelvic structures. Healthcare providers may opt for cesarean delivery to mitigate risks (43). This observation is consistent with a study conducted in Japan (33) but contrasts with studies from Italy (39) and North Macedonia (24). Higher cesarean section rates among adolescent mothers in this study and Japan may result from healthcare priorities. Japan's healthcare system may emphasize precautionary cesareans due to concerns about adolescents’ physical immaturity and labor risks. In contrast, Italy and North Macedonia favor vaginal deliveries unless medically necessary. Differences in labor complications, antenatal care quality, socioeconomic factors, and study designs may also contribute to these variations.

Major perineal tears were significantly more common among the adolescent mothers (8.6%) than the adult mothers (3.3%), nearly three times higher, consistent with findings from Eritrea (31). This may result from less mature pelvic structures, labor complications, increased instrumental deliveries, limited prenatal care, poor nutrition, and inadequate obstetric care, compounded by physical and emotional immaturity during delivery (44).

In conclusion, our study reveals a significantly higher risk of adverse maternal outcomes among adolescent mothers, emphasizing the importance of tailored prenatal care, enhanced access to quality health services, and education for this group. Notably, the likelihood of adverse outcomes decreases by approximately 50% with each additional year of adolescence, highlighting better outcomes for older adolescents (18, 19) compared to those under 18. Although our study grouped adolescents (10–19 years) for comparison with adults, future research should stratify by age to identify specific risks. Policies should prioritize preventing adolescent pregnancies through comprehensive sexual intercourse education, access to contraception, and strengthened youth-friendly health services. Further studies are needed to investigate the causes of these risks and design effective interventions.

## Limitations of the study

This study was presented based on hospital birth data, which cannot be generalized to the community since many adolescent mothers may be unable to deliver in a hospital due to a lack of awareness, poverty, or neglect by family or their community. In such scenarios, the birth process and outcomes could be more adverse than this result has shown. Therefore, larger community-based studies are needed.

## Conclusion

This study highlights significant disparities in maternal outcomes between adolescent and adult mothers, with adolescents facing higher rates of complications such as anemia, preterm labor, cesarean delivery, and pregnancy-induced hypertension. These findings emphasize the need for targeted interventions to address both medical and sociodemographic challenges. Key strategies include incorporating comprehensive sexual intercourse education to promote safe sex practices, providing easy access to affordable contraception to prevent unplanned pregnancies, and establishing youth-friendly services at public health centers that offer tailored care and confidential support. Mental health support and social programs, such as peer groups and parenting classes, are essential for helping adolescent mothers manage stress and early motherhood. Policies should focus on improving access to prenatal care, reducing socioeconomic barriers, and supporting adolescent mothers’ needs. Further, community-based research is needed to better understand the causes of adverse maternal outcomes and to develop effective intervention strategies.

## Data Availability

The original contributions presented in the study are included in the article/Supplementary Material, further inquiries can be directed to the corresponding author.
